# Life satisfaction and mental health among transgender students in Norway

**DOI:** 10.1186/s12889-020-8228-5

**Published:** 2020-01-30

**Authors:** Norman Anderssen, Børge Sivertsen, Kari Jussie Lønning, Kirsti Malterud

**Affiliations:** 1 Department of Psychosocial Science, Faculty of Psychology, University of Bergen, Bergen, Norway; 2 Research Unit for General Practice, NORCE Norwegian Research Centre, Bergen, Norway; 3 Department of Health Promotion, Norwegian Institute of Public Health, Bergen, Norway; 4 Department of Research & Innovation, Helse Fonna HF, Haugesund, Norway; 5 Department of Mental Health, Norwegian University of Science and Technology, Trondheim, Norway; 6 The Norwegian Medical Association, Oslo, Norway; 7 The Student Welfare Organization of Oslo and Akershus (SiO), Oslo, Norway; 8 Department of Global Public Health and Primary Care, University of Bergen, Bergen, Norway; 9 The Research Unit and Section of General Practice, Department of Public Health, University of Copenhagen, Copenhagen, Denmark

**Keywords:** Transgender, Binary, Non-binary, Gender incongruence, Gender minority, Mental health, Suicidal behavior, Loneliness, Students

## Abstract

**Background:**

Social attitudes to transgender persons and other gender minorities vary around the world, and in many cultures, prejudices and social stigma are common. Consequently, transgender persons face challenges related to discrimination and negative attitudes among the public. The purpose of this study was to compare life satisfaction, loneliness, mental health, and suicidal behavior among transgender students with cisgender students’ experiences in a nationwide sample of Norwegian students pursuing higher education.

**Methods:**

In total,50,054 full-time Norwegian students completed an online questionnaire (response rate 30.8%), of whom 15,399 were cisgender males, 34,437 cisgender females, 28 individuals who reported being binary transgender (12 transwomen and 16 transmen), and 69 individuals non-binary transgender persons. The measures included questions concerning gender identity, life satisfaction (Satisfaction With Life Scale), loneliness (The Three-Item Loneliness Scale), mental health problems (Hopkins Symptoms Check List), mental disorders, and suicidal ideation, suicidal behavior, and self-harm. Chi-square tests, Independent-Samples Kruskal-Wallis tests, and logistic regression analyses were used to examine differences between gender identities.

**Results:**

Transgender students reported significantly more psychosocial burdens on all measures. There were no significant differences in any of the measures between the binary and non-binary transgender students.

**Conclusion:**

The findings call for increased awareness about welfare and health for transgender students in Norway. Higher education institutions need to consider measures at various levels to establish a learning environment that is more inclusive for gender minorities.

## Background

*Transgender* persons include different groups of individuals who are *gender incongruent,* with their identities or expressions of gender not matching the sex they were assigned at birth [1, 2]. *Cisgender persons*, on the other hand, comprise the social majority, whose gender identities or expressions are congruent with their sex assigned at birth. Social attitudes to gender incongruence and non-conformity with societal expectations vary around the world, and in many cultures, prejudices and social stigma are common. Consequently, transgender persons face challenges related to discrimination [3] and negative attitudes among the public [4], and discrimination and stigma of gender minorities is found to be associated with negative mental health outcomes in these groups [5].

There are conceptual concerns with the terminology used for gender minorities. One subgroup among transgender persons comprises individuals who fulfilled the previous diagnostic criteria for *transsexualism* in the International Classification of Diseases and Related Health Problems version 10 (ICD-10)*,* many appearing in statistics for gender affirmation care at specialist clinics [1]. However, a substantial proportion of gender incongruent persons neither classify themselves as transsexuals nor accept categorization as having a mental disorder. Consequently, the new ICD-11 no longer classifies gender incongruence in the chapter of mental and behavioral disorder but in the chapter of conditions relating to sexual health [6]. Transgender persons may see themselves as binary or non-binary: *Binary* means identifying as either a man or a woman, such as a transgender female or male, while *non-binary* implies a dismissal of the dual gender model. *Gender dysphoria* is “broadly defined as discomfort or distress that is caused by a discrepancy between a person’s gender identity and that person’s sex assigned at birth (and the associated gender role and/or primary and secondary sex characteristics)” (p. 166) [1]. Such dysphoria may or may not occur among transgender persons, leading to a range of differentiated needs and wish for health care services regarding, for example, support, hormone therapy, or surgery. This terminology is currently fluid and evolving, and there “may be substantial variations in meaning and interpretation of various terms depending on the individual person, context, and culture.” (p. 2392) [2].

Gender minorities have become increasingly visible in the Scandinavian countries, and concerns have been raised regarding the health and welfare of these groups. Epidemiological research on gender minorities is limited, however. Studies are few and primarily based on convenience samples. Still, there are substantial indications that many transgender persons, including youths, struggle with psychosocial issues. In a review of 15 studies published between 2011 and 2016, Connolly et al. (2016) demonstrated that transgender youths struggle more often with depression, self-harm, and suicide behaviors than do cisgender youths [7]. A recent population-based study among 131,901 US high school students in ten states and nine urban school districts revealed that transgender students were at a higher risk of victimization, substance use, and suicidal behaviors than were cisgender males [8]. A study from Minnesota including more than 88,000 students (9th and 11th grades) showed that transgender and gender incongruent individuals reported life time suicidal ideation (61.3%) and life time suicide attempts (31.0%) more often than did their cisgender peers (20.0 and 7.1%, respectively) [9]. Similar findings were reported in studies based on convenience samples from the US and Canada [10, 11]. Studies based on convenience samples of transgender persons from Finland [12] and Sweden [13] demonstrated higher proportions of depression and anxiety compared to cisgender persons, especially among the youngest participants. Still, a small but growing body of studies indicates that many transgender persons report standards of living and mental health equivalent to the population as a whole [14–16].

On the basis of the above, we conclude that there is an urgent need to address the welfare and health of transgender groups and individuals, including young people. The aim of the present study was to compare life satisfaction, loneliness, mental health, and suicidal behavior among transgender students with cisgender students.

## Methods

### Procedure

The SHoT2018 study (Students’ Health and Wellbeing Study) is a national student survey for higher education in Norway, initiated by the three largest student welfare organizations (Sammen [Bergen and surrounding area], SiT [Trondheim and surrounding area], and SiO [Oslo and Akershus]). The data for the SHoT2018 was collected electronically through a web-based platform. Details of the study have been published elsewhere [17], but in short, the SHoT2018 was conducted between February 6 and April 5, 2018, having invited all full-time Norwegian students pursuing higher education (both in Norway and abroad) to participate. In all, 162,512 students fulfilled the inclusion criteria, of whom 50,054 students completed the online questionnaires, yielding a response rate of 30.8%.

### Ethics

The SHoT2018 study was approved by the Regional Committee for Medical and Health Research Ethics in Western Norway (no. 2017/1176). Electronic informed consent was obtained after the participants had received a detailed introduction to the study.

### Instruments

#### Gender identity

In the current study, gender identity was assessed using the question “What is your gender?” with three possible response options: “Woman,” “Man” and “Other.” If the students responded “Woman” or “Man,” they were categorized as cisgender. If the students responded “Other,” they could choose from three additional response options: “Male-to-female transgender (MtF),” “Female-to-male transgender (FtM),” and “Other, please describe your gender identity,” for which the students could answer in free text. The free-text responses were then manually categorized by two experts in the field (authors NA and KM). The large majority (*n* = 69) of all free-text responses (*n* = 87) were variations of “non-binary gender,” “gender fluid,” or “agender,” and for the purposes of the present study, these responses were combined into one category (labeled “non-binary gender”). The remaining responses (*n* = 18) were coded as “other,” because of non-relevant responses that we could not categorize properly (e.g. “helicopter”), and they were omitted from further analysis. Since we did not ask about sex assigned at birth, we cannot know if the cisgender groups included persons who today identify different from the sex they were assigned at birth. With this limitation in mind, and due to small cells/challenges related to lack of statistical power, the following gender categories are used throughout the current paper: 1) cisgender male (*n* = 15,399), 2) cisgender female (*n* = 34,437), 3) binary transgender female (*n* = 12) or male (*n* = 16), and 4) non-binary transgender (*n* = 69).

#### Life satisfaction

Life satisfaction was assessed by the Satisfaction With Life Scale (SWLS) [18]. The SWLS is a 5-item scale designed to measure global cognitive judgments of one’s life satisfaction (not a measure of either positive or negative affect). Participants indicate how much they agree or disagree with each of the five items using a 7-point scale that ranges from 1 (strongly disagree) to 7 (strongly agree). In the current study, the SWLS was analyzed in three ways: 1) as a continuous total score (range 5–35), 2) using pre-defined categories (*dissatisfied*: total SWLS score 5–19; *neutral*: total SWLS score 20–25, and *satisfied*: total SWLS score 26–35), and 3) dichotomously, using a total SWLS total score of < 19 as the cut-off value indicating poor life satisfaction. The Cronbach’s alpha of the SWLS in the current study was 0.89. Comparisons of the SWLS in student populations in 42 countries have shown good psychometric properties [19].

#### Loneliness

Loneliness was assessed using an abbreviated version of the widely used UCLA Loneliness Scale, “The Three-Item Loneliness Scale” (T-ILS) [20]. The instrument includes the following three items, “How often do you feel that you lack companionship?”; “How often do you feel left out?”; and “How often do you feel isolated from others?”, with the five response options: “never,” “seldom,” “sometimes,” “often,” and “very often.” The T-ILS has displayed satisfactory reliability and both concurrent and discriminant validity in two US nationally representative population-based studies [20]; it has also performed well among US college students [21]. The three items were analyzed separately, and each item was dichotomized using “often” or “very often” as the cut-off value. The Cronbach’s alpha of the T-ILS in the current study was 0.88.

#### Mental health problems

Mental health problems were assessed using the Hopkins Symptoms Checklist (HSCL-25) [22], derived from the 90-item Symptom Checklist (SCL-90), a screening tool designed to detect symptoms of anxiety and depression. The HSCL-25 is composed of a 10-item subscale for anxiety and a 15-item subscale for depression, with each item scored on a Likert scale from 1 (“not at all”) to 4 (“extremely”). The period of reference is the preceding 2 weeks. The HSCL-25 has demonstrated good psychometric properties [23], and a recent study showed that a uni-dimensional model is most appropriate for HSCL-25 in a student population [24]. The Cronbach’s alpha of the HSCL-25 in the current study was 0.95. An average score on the HSCL-25 ≥ 2.0 is commonly used as a conservative cut-off for identifying a high level of depressive and anxiety symptoms. In the current study, the HSCL-25 was analyzed both as a continuous average score (range 1–4) and using pre-defined categories (low: average HSCL-25 score < 1.75; moderate: average HSCL-25 score ≥ 1.75 < 2.0; and high: average HSCL-25 score ≥ 2.0).

#### Mental disorders

Mental and somatic conditions/disorders were assessed by self-report using a pre-defined list adapted to fit this age cohort. The list was based on a similar operationalization used in previous large population-based studies (the HUNT studies) and included several subcategories for most conditions/disorders (not listed here) [25]. Only mental disorders were included in the current study, and the list comprised the following specific disorders/group of disorders: attention deficit hyperactivity disorder (ADHD), anxiety disorder, autism/Asperger’s, bipolar disorder, depression, posttraumatic stress disorder (PTSD), schizophrenia, personality disorder, eating disorder, Tourette’s syndrome, obsessive compulsive disorder (OCD), and other (free text). Answering “yes” to any of these conditions was coded as the presence of a mental disorder. No analyses of specific disorders were conducted due to small cell sizes in some of the gender-identity groups.

#### Suicidal ideation, suicidal behavior, and self-harm

History of suicidal ideation, suicide attempts and self-harm were assessed with three items drawn from the Adult Psychiatric Morbidity Survey (APMS) [26]; “Have you ever seriously thought of taking your life, but not actually attempted to do so?”; “Have you ever made an attempt to take your life, by taking an overdose of tablets or in some other way?”; and “Have you ever deliberately harmed yourself in any way but not with the intention of killing yourself (i.e., self-harmed)?” respectively. The questions about self-harm thoughts were adapted from the Child and Adolescent Self-harm in Europe study (CASE) [27]: “Have you ever seriously thought about trying to deliberately harm yourself but not with the intention of killing yourself but not actually done so?” (A response of yes or no was possible.) If respondents confirmed any item, timing of the most recent episode, frequency of episodes, and age at first onset were then assessed, but these were not included in the current study due to small cell sizes in some of the gender-identity groups. More details on suicidal ideation in the SHoT2018 study have been published elsewhere [28].

#### Demographic information

All participants indicated their age. Economic activity was coded dichotomously according to self-reported annual income (before tax and deductions, and not including loans and scholarships): “economically active” (annual income > 10,000 Norwegian Krone (NOK)) versus “economically inactive” (< 10,000 NOK). The reason for excluding loans and scholarships from annual income was that all students taking higher education in Norway receive near-identical loans/scholarships, and in this respect, we were more interested in students earning additional money from working while being a full-time student. Students living abroad indicated their current country of residence, which was subsequently categorized by continent. Finally, a participant was classified as an immigrant if either the student or his/her parents were born outside Norway.

### Statistics

IBM SPSS version 25 (SPSS Inc., Chicago, IL, USA) for Mac was used for all analyses. Chi-square tests and Independent-Samples Kruskal-Wallis tests were used to examine differences between the four gender-identity groups (cisgender male, cisgender female, binary transgender, and non-binary transgender) in quality of life, mental health, mental disorders, self-harm/suicidal ideation, and loneliness. We tested for pairwise comparisons of proportions between the gender groups by employing the “Compare column proportions” function available for Chi-square tests in SPSS. Logistic regression analyses were conducted to provide effect-size estimates (odds ratios [ORs]) on the same outcomes between binary transgender and non-binary transgender categories, using cisgender male and female gender combined as the reference group. The normality of the data was examined using skewness and kurtosis, and all continuous measures (HSCL-25 and SWLS) were well within the recommended ranges (+/− 2) [29]. There was generally little missing data (*n* < 270 [0.5%]), and hence missing values were handled using listwise deletion. As the SHoT2018 study had several objectives and was not designed to be a study of transgender students specifically, no a priori power calculations were conducted to ensure that the sample size had sufficient statistical power to detect differences in outcomes.

## Results

### Sample characteristics

In all, 115 individuals reported a gender identity other than male (*n* = 15,399) or female (*n* = 34,437). Of these, 28 individuals reported being binary transgender female (*n* = 12) or male (*n* = 16), while 87 individuals reported themselves as “other.” Of these, the large majority (*n* = 69) were classified as non-binary transgender persons. Table 1 details the demographic characteristics of the gender identity groups and total sample. There were no significant differences between transgender individuals and cisgender males and females in terms of age, immigrant status, or country of residence (continent). However, transgender individuals were more likely to be economically inactive (χ^2^ [df = 3, *N* = 48,216] = 261.4, *P* < .001).

**Table 1:** Demographic characteristics of the sample

	Cisgender male	Cisgender female	Binary transgender	Non-binary transgender	Total sample
Age, mean (SD)	23.5 (3.3) _a*_	23.1 (3.3) _b_	24.0 (4.1) _a,b_	23.2 (3.4) _a,b_	23.2 (3.3)
Economically inactive (< 10 K NOK), % (n)	15.8% (*n* = 2387) _a_	11.6% (*n* = 3833) _b_	61.5% (*n* = 16) _c_	40.0% (*n* = 26) _c_	13.0% (*n* = 6262)
Immigrant, % (n)	8.2% (*n* = 1262) _a_	7.9% (*n* = 2726) _a_	3.6% (*n* = 1) _a_	10.1% (*n* = 7) _a_	8.0% (*n* = 3996)
Country/continent of residence, % (n)					
Norway	99.3% (n = 15,077) _a_	99.1% (*n* = 33,687) _b_	96.3% (n = 26) _a,b_	100.0% (n = 69) _a,b_	99.1% (n = 48,859)
Asia	0.2% (n = 26) _a_	0.2% (*n* = 74) _a_	n/a	n/a	0.2% (*n* = 100)
Africa	0.1% (n = 8) _a_	0.1% (*n* = 36) _a_	3.7% (n = 1) _b_	n/a	0.1% (*n* = 45)
North America	0.2% (*n* = 32) _a_	0.2% (*n* = 73) _a_	n/a	n/a	0.2% (*n* = 105)
South America	0.1% (n = 12) _a_	0.1% (n = 18) _a_	n/a	n/a	0.1% (*n* = 30)
Oceania	0.2% (n = 30) _a_	0.3% (*n* = 116) _b_	n/a	n/a	0.3% (*n* = 146)

* Significant gender group differences are indicated for each row using subscript letters, calculated at the 0.05 significance level

n/a = not applicable (empty cell)

### Life satisfaction

**Fig. 1:**
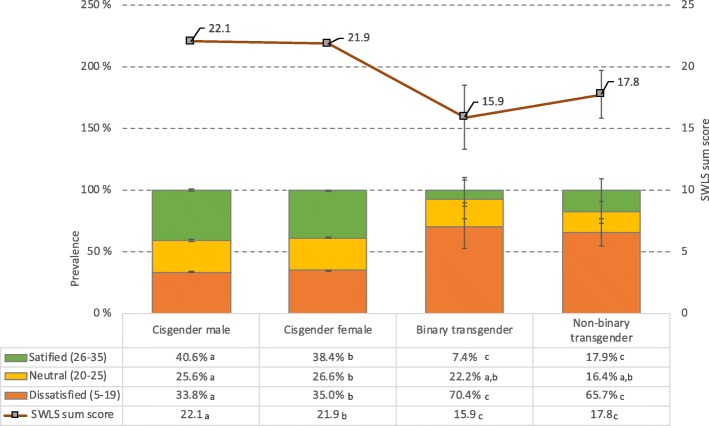
Prevalence of poor life satisfaction (in bars) stratified by gender identity. Lines indicate continuous SWLS sum scores. Error bars represent 95% confidence intervals. SWLS=Satisfaction With Life Scale. Significant gender group differences are indicated for each row in the table using subscript letters, calculated at the .05 significance level

Transgender individuals reported significantly lower life satisfaction than did cisgender individuals. Figure 1 displays the SWLS scores both in categories and continuously. Results from the Chi-square tests showed that 70% of binary transgender and 64% of non-binary transgender individuals reported being dissatisfied with their lives (SWLS< 19), compared to 34–35% among cisgender individuals (χ^2^ (df = 6, N = 48,514) = 65.8, P < .001). The Independent-Samples Kruskal-Wallis test showed that transgender individuals also scored low on the SWLS total score (binary transgender: 15.9 [SD = 6.5] and non-binary transgender: 17.5 [SD = 7.2]) and significantly lower than both cisgender males (22.1 [SD = 6.8]) and cisgender females (21.9 [SD = 6.7]); all Ps < .001). There were no significant differences in life satisfaction (categorically or continuously) between binary and non-binary transgender individuals (see Fig. 1 for details).

As detailed in Table 2, the logistic regression analysis showed that the OR of reporting lower life satisfaction was 4.48 (95% confidence interval [CI] 1.96–13.38) for transgender binary individuals compared to cisgender males and females, and a similar association was found for non-binary transgender individuals (OR = 3.32 [95% CI 2.03–5.43]). The ORs were somewhat reduced but remained statistically significant, when adjusting for potential confounders (age, income/economic activity, country of residence, and immigrant status).

**Table 2:** Odds ratios (ORs) of poor outcomes in transgender and non-binary gender compared to cisgender males and females

	Cisgender male or female gender	Binary transgender		Non-binary transgender	
	Unadjusted model	Unadjusted model	Adjusted model^*^	Unadjusted model	Adjusted model^*^
Outcome variable	OR (95% CI)	OR (95% CI)	OR (95% CI)	OR (95% CI)	OR (95% CI)
Poor life satisfaction (SWLS< 19)	1.00 (ref)	4.48 (1.96–10.24)	3.78 (1.56–9.15)	3.61 (2.18–5.98)	3.12 (1.82–5.33)
Loneliness (“often” or “very often” versus “never” or “seldom”)					
Lack companionship	1.00 (ref)	4.42 (1.70–11.51)	3.05 (1.12–8.32)	2.69 (1.50–4.84)	2.12 (1.13–3.95)
Left out	1.00 (ref)	4.64 (1.76–12.18)	3.20 (1.18–8.87)	5.25 (3.04–9.06)	4.20 (2.33–7.57)
Isolated from others	1.00 (ref)	6.26 (2.27–17.23)	3.58 (1.23–10.47)	5.82 (3.32–10.22)	4.35 (2.35–8.05)
Mental health problems (HSCL-25 > 2.00)	1.00 (ref)	2.75 (1.31–5.78)	2.48 (1.12–5.46)	4.63 (2.82–7.59)	4.07 (2.41–6.87)
Mental disorder (any)	1.00 (ref)	7.29 (3.45–15.41)	5.51 (2.32–11.43)	8.50 (5.24–13.79)	6.55 (3.92–10.95)
Self-harm (lifetime)	1.00 (ref)	5.47 (2.59–11.57)	4.45 (2.01–9.86)	4.74 (2.95–7.62)	4.46 (2.69–7.40)
Self-harm thoughts (lifetime)	1.00 (ref)	4.56 (2.16–9.64)	4.53 (2.02–10.14)	4.72 (2.92–7.61)	3.50 (2.11–5.81)
Suicide attempt (lifetime)	1.00 (ref)	6.23 (2.52–15.39)	5.56 (2.20–14.07)	6.90 (3.94–12.09)	6.12 (3.34–11.20)
Suicide thoughts (lifetime)	1.00 (ref)	6.79 (3.13–14.71)	5.27 (2.32–11.98)	6.24 (3.83–10.16)	5.26 (3.26–8.84)

* Adjusted for age, income/economic activity, country of residence, and immigrant status

*CI* = Confidence interval; *SWLS*=Satisfaction With Life Scale; HSCL-25 = Hopkins Symptoms Checklist-25

### Loneliness

**Fig. 2:**
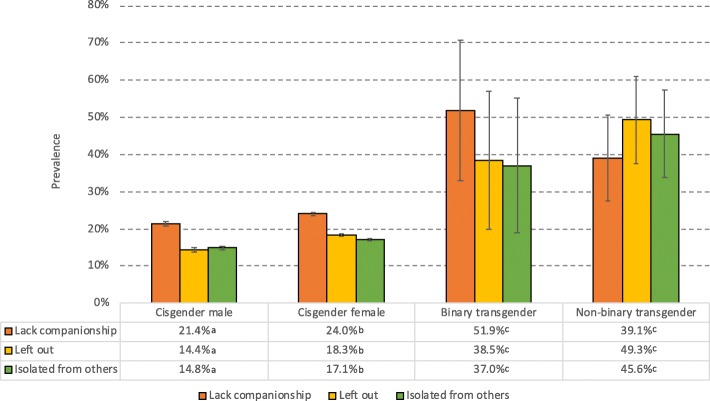
Prevalence of loneliness (“often” or “very often”) stratified by gender identity. Error bars represent 95% confidence intervals. Significant gender group differences are indicated for each row in the table using subscript letters, calculated at the .05 significance level

Binary and non-binary transgender individuals also reported substantially more loneliness than did cisgender males and females. As detailed in Fig. 2, the Chi-square tests showed that 38–52% of binary transgender individuals reported often or very often either “lacking companionship,” “feeling left out” or “feeling isolated from others,” and similar rates were observed for non-binary transgender individuals (38–48%). The corresponding rates for cisgender males and females were 15–21% and 17–24%, respectively. Results from the logistic regression analysis showed that the OR of reporting “often” or “very often” on one of the three loneliness items was 4.02 (95% CI 1.84–8,78) for binary transgender individuals compared to cisgender males and females, while the OR for non-binary transgender individuals was 2.72 [95% CI 1.71–4.34]). As detailed in Table 2, similar ORs were found for all the three loneliness variables, with the highest OR being observed for “Isolated from others” (see Table 2 for details). The associations remained significant in the adjusted analysis, although the ORs were somewhat reduced (see Table 2 for details).

### Mental health problems

**Fig. 3:**
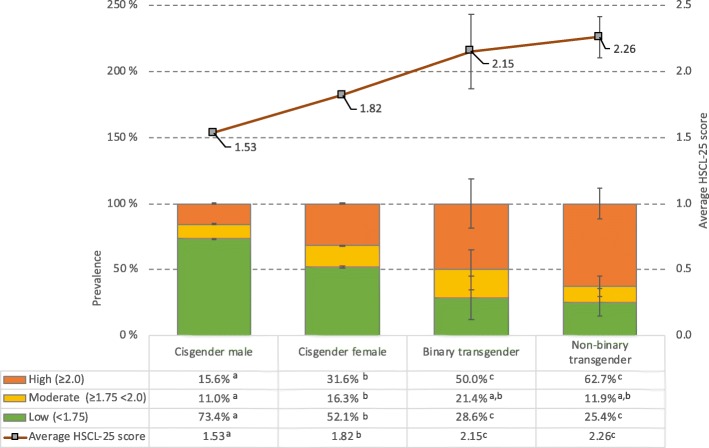
Prevalence of mental health problems (in bars) stratified by gender identity. Lines indicate HSCL average scores. Error bars represent 95% confidence intervals. HSCL-25 = Hopkins Symptoms Checklist-25. Significant gender group differences are indicated for each row in the table using subscript letters, calculated at the .05 significance level

Mental health problems were significantly more frequently reported among transgender individuals than among cisgender males and females. As displayed in Fig. 3, 50.0 and 62.7% of binary and non-binary transgender individuals, respectively, scored over the cut-off of 2.0 on the HSCL-25, indicating a high level of anxiety and depression symptoms. In comparison, 15.6% of cisgender males and 31.6% of cisgender females scored over this cut-off (χ^2^ [df = 6, *N* = 49,825] = 2080, *P* < .001). Binary and non-binary transgender individuals also had a significantly higher average HSCL score (2.15 [SD = 0.73] and 2.26 [SD = 0.64], respectively), than both cisgender males (1.53 [SD = 0.48] and females 1.82 [SD = 0.56]; all Ps < .001).

As also detailed in Table 1, the OR of reporting a high level of mental health problems was 2.75 (95% CI 1.31–5.75) for binary transgender males or females compared to cisgender peers, and an even stronger association was observed for non-binary transgender individuals (OR = 4.63 [95% CI 2.82–7.59]). The ORs were only slightly reduced and remained significant after adjustment for confounders.

### Mental disorder

Fifty-seven percent and 59% of binary and non-binary transgender individuals, respectively, reported having a mental disorder. In comparison, 18% of cisgender females and 11% of cisgender males reported this (χ^2^ [df = 3, *N* = 49,933] = 560.7, *P* < .001). The OR of reporting a mental disorder was 7.29 (95% CI 3.45–15.41) for binary transgender individuals compared to cisgender males and females, and a similarly strong association was observed for non-binary transgender individuals (OR = 8.50 [95% CI 5.24–13.79]). The ORs remained significant in the adjusted analysis.

### Self-harm, self-harm thoughts, suicide attempts, and suicide thoughts

**Fig. 4:**
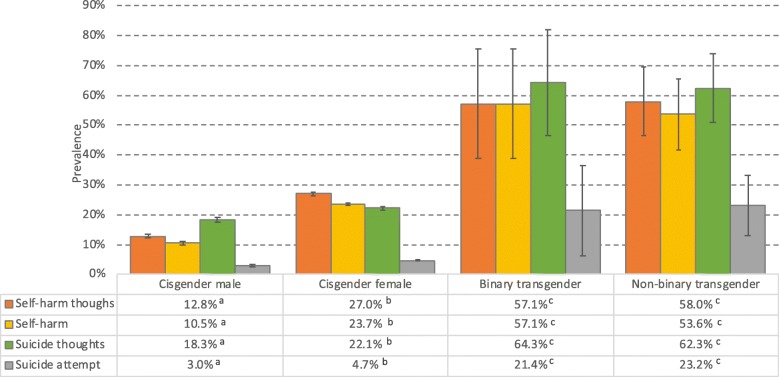
Prevalence of lifetime self-harm, self-harm thoughts, suicide attempts, and suicide thoughts, stratified by gender identity. Error bars represent 95% confidence intervals. Significant gender group differences are indicated for each row in the table using subscript letters, calculated at the .05 significance level

Self-harm and suicidal ideation were significantly more common among binary and non-binary transgender individuals than among cisgender males and females. Figure 4 shows the prevalence of all four self-harm and suicidal ideation variables. The prevalence of lifetime self-harm and self-harm thoughts ranged from 54 to 58% in both transgender and non-binary individuals, compared to 11–13% in cisgender males and 24–27% in cisgender females (all Ps < .001). The corresponding ORs for self-harm and self-harm thoughts ranged from 4.6 to 5.5 for both transgender and non-binary individuals (see Table 2 for details) compared to cisgender males and females. Similar patterns were observed for both suicide attempts and suicidal thoughts, with substantially higher prevalences among both binary and non-binary transgender individuals than among cisgender individuals (see Fig. 4 for details). The ORs remained significant in the adjusted analysis, although the ORs were slightly reduced (see Table 2 for details).

## Discussion

On the basis of the data from a national student survey for higher education in Norway (the SHoT2018 study), we compared life satisfaction, loneliness, mental health, and suicidal behavior among transgender students (*N* = 96) with cisgender students (*N* = 49,836). Many transgender students reported experiences in the same range as their cisgender peers, but on average, they scored in the direction of psychosocial hardship. Compared to cisgender students, transgender students more often reported psychosocial burdens regarding satisfaction with life, loneliness, mental health problems and disorders, and suicide-related measures. There were no significant differences in any of the measures between the binary and non-binary transgender students. These findings are in line with studies among transgender youth in other countries [7, 9–12, 30], indicating a vulnerable gender minority population group in need of special attention in Norway as well.

### The costs of violating gender normativity

We believe that the increased burdens reported by transgender students in Norway are associated with violating existing gender norms—not only the norms for appropriate behaviors for men and women but also the cisnormative notion that only two reciprocally exclusive gender categories of men and women exist. In Norway and most other industrialized countries, these norms permeate all aspects of society, including people’s identities and behaviors, and are taken for granted by lay persons, health professionals, and educators [31]. Each young transgender person faces the personal challenges and costs of violating these norms. They run the continuous risk of being discriminated against and ridiculed.

Identity theory and life course studies see this period as crucial with regard to, for example, intimacy and sexuality, personal identity and taste, group belonging, and dealing with new types of interpersonal relationships and professional standards [32, 33]. For many transgender persons, gender-identity concerns arise during the formative years of adolescence and young adulthood, corresponding to the age period of higher education. Transgender students face unique challenges related to the defining nature of their identities, appropriating their personal way of behaving and presenting themselves in line with their gender identity, and learning to deal with possible body changes and medical treatment, as well as the demanding work of coming out and responding to possible prejudice stigmatization. They have to face issues such as: “What is my gender identity and how do I manage and navigate in this field?” and “Should I tell friends and relatives?” In this more challenging and complex landscape of personal and interpersonal concerns, the young transgender student must navigate and find solutions.

In Norway, the last decade has seen signs of more differentiated gender conceptualizations. For example, transgender persons are more visible in the media, there are public and academic discussions about diagnoses such as gender dysphoria and treatment options, activist organizations include various transgender groups, and the Norwegian government now utilizes the “LGBTQI” phrase (Lesbian, Gay, Bisexual, Transgender, Queer, and Intersex) in official documents [34]. In addition, public attitudes in Norway are gradually becoming less negative toward transgender persons, as revealed by population-based surveys in 2008, 2013, and 2017 [35]. Even so, transgender persons in Norway report experiences in school (e.g., a lack of information about different gender identities) and the health system (e.g., a lack of adequate counseling for transgender patients) indicating the need for improvements [36].

### Gender incongruence vs gender dysphoria

The psychosocial burdens of many transgender students represent a complex mixture of external social attitudes to gender non-conformity and internal emotional reactions to gender non-congruence, which are probably differently distributed within the sample. For some transgender persons the aspect of gender dysphoria may be most prominent, leading to serious bodily dissatisfaction and a subsequent strong wish for medical gender affirmation. Identifying and being recognized as a patient in need of medical treatment may enhance emotional coping, whereas being dismissed by the health care system may represent a major existential threat to identity. However, not all transgender individuals desire or need medical gender affirmation. Distress arises when the need for is greater than access to gender affirmation. For those binary transgender persons who are not visibly transgender, negative social responses to gender incongruence may be reduced because public attitudes are less negative toward binary transgender persons than toward non-binary transgender persons [35].

For other transgender persons, gender dysphoria may possibly be more associated with social role than with bodily attributes. By not aspiring to pass as a cisgender male or female, the non-binary transgender person may appear more provocative to cultural cis-normativity, given the finding noted above that more Norwegians hold negative attitudes toward gender-fluid persons than toward persons who have received gender-confirming medical treatment [35], increasing the risk of social sanctions. Importantly, many transgender persons manage well, as documented in a recent survey in Belgium [16], suggesting that there are important resilience factors that need to be explored in future studies.

### The need for health-promoting efforts for transgender students

The present findings imply that a range of health-promoting efforts for transgender students is needed. Strategies for societal changes in the direction of greater acceptance of gender diversity are vital. Here, we want to point to the specific situation for various transgender students in higher education. A qualitative study from the US identified four factors relating to the well-being and safety of transgender students: 1) coming out as transgender in the classroom, 2) interactions with fellow students and interactions with instructors, 3) course context (e.g., online or not; in online courses, one may have less control over exposure as a transperson due to university policies regarding legal names and email), and 4) campus experiences [37]. On the basis of these arenas for possible interventions, the current findings and those of other studies (e.g., Swanbrow Becker et al.) [11], and our knowledge of higher institutions, we advise higher education institutions in Norway to establish a learning climate that is more inclusive for gender minorities at the policy and practical levels. For example, the institutions can emphasize the responsibility of instructors to create safe environments in which students can openly express gender diversity, that instructors should respect students’ chosen name, and that instructors should always behave as if gender diversity exists in student groups. In addition, the institutions can follow a policy of non-tolerance of harassment. A visible sign of institutional support to transgender students can be to introduce non-gendered bathroom facilities all over campus.

Another field relates to course content. At the very least, transgender students should be mentioned in diversity programs. We also advise that students and health and social welfare institutions—such as general practitioners, student health services, and student welfare organizations—be routinely advised that many gender minority students face important psychosocial challenges and that opportunities for receiving help exist. Counseling services should be aware of heightened risk of trauma history with harassment and victimization among transgender students. After implementing transgender-promoting interventions, varying from attitude changes among students and instructors to institutional policies, a well-functioning institution would also, as a routine, evaluate interventions.

### Strengths and limitations

An important strength of this study is the population-based nationwide sample with an acceptable response rate and thus, the sample provides data with the potential for generalizability. Generalizations from the transgender sample should still be made with caution, however, because we have no information with regard to how many among these groups decided not to participate in the study or how well the gender questions differentiated between relevant groups. An additional strength is that responses from transgender participants were directly comparable to those from other students, since recruitment and information given about the survey was identical for all students.

One limitation is that we did not ask for measures of gender dysphoria or medical gender affirmation. Furthermore, we did not ask about sex assigned at birth, and may therefore not have identified all students who identify as other than the gender they were assigned at birth. Nonetheless, we were able to compare binary and non-binary respondents within the transgender group through the analyses. Although no significant differences were identified in these analyses, we call attention to samples and analyses even more attentive to subgroups among transgender youth that may be specifically vulnerable or resilient. A final limitation is the small sample size of the transgender students included, which is reflected by the wide confidence intervals. The small group sizes also mean that we had insufficient statistical power to detect potential differences between binary and non-binary transgender persons.

## Conclusion

The findings clearly indicate that transgender students should receive attention to prevent social and emotional hardships and to promote their health. Our findings call for increased awareness of welfare and health for gender minority students in Norway. That said, to provide a nuanced picture and to avoid reinforcing stereotypes, we also want to point out that many transgender students manage to navigate challenging interactional processes well, possibly due to personal and social resilience factors. Furthermore, substantial proportions of transgender students report well-being and mental health status comparable to those of cisgender students. The analyses do not give specific indications about which efforts should be implemented by higher education institutions, but a general ambition should be to establish a learning environment that is more inclusive for gender minorities.

## Data Availability

The SHoT2018 dataset is administrated by the National Institute of Public Health. Approval from a Norwegian regional committee for medical and health research ethics [https://helseforskning.etikkom.no] is a pre-requirement. Guidelines for access to SHoT2018 data are found at [https://www.fhi.no/en/more/access-to-data].

## Abbreviations

APMS: Adult Psychiatric Morbidity Survey
CASE: Child and Adolescent Self-harm in Europe study
HSCL-25: The Hopkins Symptoms Checklist (25 item version)
HUNT: Helseundersøkelsen i Nord-Trøndelag (Health Survey of North Trøndelag)
SHoT2018: Students’ Health and Wellbeing Study, 2018
SiO: Studentsamskipnaden i Oslo (Student Welfare Organization at University of Oslo)
SiT: Studentsamskipnaden i Tromsø (Student Welfare Organization at University of Tromsø)
SWLS: Satisfaction With Life Scale
T-ILS: The Three-Item Loneliness Scale
